# A Study on Mediation by Offspring BMI in the Association between Maternal Obesity and Child Respiratory Outcomes in the Amsterdam Born and Their Development Study Cohort

**DOI:** 10.1371/journal.pone.0140641

**Published:** 2015-10-20

**Authors:** Margreet W. Harskamp-van Ginkel, Stephanie J. London, Maria C. Magnus, Maaike G. Gademan, Tanja G. Vrijkotte

**Affiliations:** 1 Department of Public Health, Academic Medical Center, P.O. Box 22660, 1100 DD Amsterdam, The Netherlands; 2 Epidemiology Branch and Laboratory of Respiratory Biology, National Institute of Environmental Health Sciences, National Institutes of Health, Department of Health and Human Services, Research Triangle Park, Durham, North Carolina, United States of America; 3 Department of Chronic Diseases, Division of Epidemiology, Norwegian Institute of Public Health, Oslo, Norway; Yale School of Public Health, UNITED STATES

## Abstract

**Background:**

A causal relationship between maternal obesity and offspring asthma is hypothesized to begin during early development, but no underlying mechanism for the found association is identified. We quantitatively examined mediation by offspring body mass index (BMI) in the association of maternal pre-pregnancy BMI on risk of asthma and wheezing during the first 7–8 years of life in a large Amsterdam born birth cohort.

**Methods:**

For 3185 mother-child pairs, mothers reported maternal pre-pregnancy BMI and offspring outcomes “ever being diagnosed with asthma” and “wheezing in the past 12 months” on questionnaires. We measured offspring height and weight at age 5–6 years. We performed a multivariate log linear regression comparing outcomes in offspring of mothers with different BMI categories. For each category we quantified and tested mediation by offspring BMI and also investigated interaction by parental asthma.

**Results:**

At the age of 7–8 years, 8% of the offspring ever had asthma and 7% had current wheezing. Maternal pre-pregnancy obesity was associated with higher risks of asthma (adjusted RR 2.32 (95% CI: 1.49–3.61) and wheezing (adjusted RR 2.16 (95% CI: 1.28–3.64). Offspring BMI was a mediator in the association between maternal BMI and offspring wheezing, but not for asthma. There was no interaction by parental asthma.

**Conclusions:**

Maternal pre-pregnancy obesity was associated with higher risks of offspring asthma and wheezing. The association between maternal obesity and offspring wheezing was both direct and indirect (mediated) through the child’s own BMI.

## Introduction

Asthma incidence in children and adults worldwide has increased dramatically in the past decades, coinciding with a steep increase of overweight and obesity [1–3]. Maternal Obesity during pregnancy is associated with an elevated risk of adverse childhood respiratory outcomes including asthma. [4–18]

A causal relationship between maternal obesity and childhood respiratory outcomes might begin in early development, either during pregnancy or childhood [12, 19–21]. Several mechanisms have been proposed and include: fetal programming [22]; common environmental and genetic factors (epiphenomena) with childhood obesity [23]; and immune modulatory effects [24]. Fetal programming describes all in utero exposures that affect gene expression and (chronic) diseases in childhood and adulthood. Diseases can originate from physiologic or metabolic adaptations that the fetus makes as a response to in utero exposures at critically sensitive periods of development [25].

Distinguishing the effects of maternal and childhood body mass index (BMI) on childhood asthma is challenging. Earlier papers investigated the mediating effect of childhood BMI by adding childhood BMI to their multivariate model and describing the attenuation of risks. Scholtens et al. concluded that the child’s birth weight and BMI at 8 years were partly mediating the effect of maternal overweight on childhood asthma [8]. Others found a minimal effect of adjustment by childhood BMI [5, 11, 15, 17]. Testing of statistical inference of the mediating effect of childhood BMI can provide new insights in the underlying mechanisms [26]. This has not been done in these prior analyses.

A relationship between maternal BMI and childhood asthma could further be affected by atopic family history. Three prior studies used stratification by parental atopy or asthma and found conflicting results [5, 8, 17]. A possible interaction could be that maternal BMI is more related to either atopic or nonatopic asthma.

The Amsterdam Born Children and Their Development (ABCD) cohort provides a unique opportunity to assess the effect of pre-pregnancy maternal BMI on ‘ever asthma’ at the age of 7–8 years in a large multi-ethnic urban birth cohort. We are the first group, using methodology described by Preacher & Hayes and MacKinnon, that formally tests the mediating effect of childhood BMI and interaction by parental asthma on the association between maternal BMI and childhood respiratory outcomes [26].

## Materials/Subjects and Methods

### Study population

This study is conducted within the ABCD cohort study (www.abcd-studie.nl). The ABCD study is a prospective community-based cohort study that examines the association between maternal lifestyle; medical, psychosocial, and environmental conditions during pregnancy; and the child’s health at birth and in later life. Details of the ABCD study design have been published [27, 28]. Approval of the study was obtained from the Central Committee on Research Involving Human Subjects in The Netherlands; the medical ethics review committees of the Academic Medical Center, Amsterdam; the VU University Medical Center Amsterdam; and the Registration Committee of the Municipality of Amsterdam. During pregnancy, all women provided written informed consent. Two weeks after the child’s fifth birthday, parents or caretakers provided written informed consent for the health check of the 5–6 year old children in the questionnaire that they received by mail. This questionnaire was only send to mothers who initially gave permission for follow-up. The informed consent procedure was approved by the committees of the Academic Medical Center, Amsterdam; the VU University Medical Center Amsterdam; and the Registration Committee of the Municipality of Amsterdam.

Between January 2003 and March 2004, all pregnant women living in Amsterdam were invited to participate in the ABCD study during their first prenatal visit to the obstetric care provider, around the 12th week of gestation. Women were asked to complete an extensive questionnaire about socio-demographic characteristics, obstetric history, lifestyle, and psychosocial conditions. The questionnaire was available in Dutch, English, Turkish, and Arabic to increase response of immigrants. Of all 12,395 pregnant women who were invited, 8266 returned the questionnaire (67% response rate).

Infant gender, birth weight and gestational age (based on ultrasound or, if unavailable (<10%), timing of the most recent menstrual period) were obtained from the Youth Health Care registration of Amsterdam’s Municipal Health Service.

Women who gave permission for follow-up received a baby-questionnaire 3 months after delivery; and a child-questionnaire when the child was 5 years and 7 years (response rate at 7–8 years = 3460, 42%). Reasons for loss to follow-up included: withdrawal from the study; infant or maternal death; unknown address; or emigration. At the age of 5 years, children were also invited for various physical measurements, including height and bodyweight (n = 3,321 children, 40%).

#### Dependent variables

The mothers reported childhood wheezing and asthma diagnosed by a physician at age 7 years on a questionnaire adapted from the International Study of Asthma and Allergies Questionnaire. Questions included: ‘Has your child had wheezing in the last 12 months?’ Has your child ever had asthma?’ ‘If so, has this been confirmed by a physician?’. We use the terms current wheezing and ever asthma for the definitions “wheezing in the last 12 months at age 7–8 years” and “ever doctor diagnosed asthma by 7–8 years” in the remaining of this paper.

### Independent variables

#### Maternal BMI before pregnancy

Mother’s BMI before pregnancy was based on self-reported weight and height from the pregnancy questionnaire (BMI = weight/(height)^2^). Weight was answered with the question: “What was your weight the last time you checked this before the pregnancy?” Measured weight and height would be preferable; however, in large epidemiological studies, self-report has been found to accurately measure BMI (intra-class correlation coefficient BMI 0.99 (95% confidence interval (CI) 0.98–0.99) [29].

BMI was used as a categorical measure in categories of lean (BMI < 18.5 kg/m^2^); normal (BMI 18.5–25 kg/m^2^), overweight (BMI 25–30 kg/m^2^), and obese (BMI ≥ 30 kg/m^2^) [30].

#### Mediating and confounding independent variables

Maternal and child characteristics evaluated as potential confounders included: maternal age (years); parity (prior birth yes or no); Western ethnicity (Dutch and other western ethnicities based on birth country mother of mother); parental asthma confirmed by physician (either maternal or paternal yes to ‘Did you ever had asthma? Has this been confirmed by a doctor?’); maternal education after primary school (low (0–6 years); middle (6–12 years); or high (12 or more years); gestational age at birth (weeks); mode of delivery (caesarean section; yes or no); child’s sex; birth weight (grams); and offspring BMI at age 5 year. BMI was calculated from height and weight (measured during well-being child visit) and normalized to z-scores using 1990 British Growth Reference [31].

Environmental exposures included: maternal smoking during pregnancy (yes vs. no); and maternal and domestic smoking at age 3 months and 5 years (yes vs. no); and duration of breastfeeding in weeks (a combined variable of maternal responses at the infant and child questionnaires and data from the Youth Health Care registration) [32].

We constructed a directed acyclic graph (DAG) to identify confounders and effect modifiers of the relationship between maternal BMI and ever asthma at age 7–8 years, using DAGitty (S1 Fig) [33]. Vectors are created for each expected effect a variable has on other variables. For example: in our cohort, ethnicity is a known predictor for offspring BMI, so a vector is pointing from ethnicity to offspring BMI [32]. Prepregnancy BMI has been associated with higher rates of pre-eclampsia and preterm birth, higher rates of caesarean section and extreme high birth weight [12]. Ethnicity and maternal education are correlated and have similar effects. The minimal adjustment set for estimating the total effect of maternal BMI on ever asthma at age 7–8 years is: non-western ethnicity; maternal age; maternal education; and parental asthma.

### Statistical analysis

The main analysis is performed as a complete case analysis. Women who returned the pregnancy questionnaire and gave birth to a live singleton child were included in the analysis. We examined demographics by maternal BMI category and by length of follow-up. We examined differences in group characteristics as a function of 4 BMI categories with the chisquare test; Kruskal Wallis test; or one-way ANOVA.

We used the defined BMI-categories to compare risk ratios (RRs), using normal BMI as a reference group in a log linear regression. For each of the associations, we analyzed a crude model first. We then included the minimal adjustment set, identified with the DAG in the first adjusted model (non-western ethnicity; maternal age; maternal education; and parental asthma). In the second adjusted model we added other independent variables: parity; smoking during pregnancy; mode of delivery; duration of breastfeeding; and domestic smoking. We chose to not control for birth weight and gestational age, as these might be in the causal pathways for the effect of maternal BMI on asthma outcome. Controlling for these would cause an underestimation of the effect of maternal BMI. We report RRs and 95% CIs.

#### Multiple imputation

We performed multiple imputations in the group of women who returned the pregnancy questionnaire and gave birth to a live singleton child, if they returned one of the two respiratory outcome questionnaires at 3 months or 7 years. We imputed all missing values (independent and dependent values), using chained equations [34]. Included variables in the imputation are: maternal height and weight; maternal education and age; ethnicity; smoking during pregnancy; maternal smoking; smoking in the house; maternal asthma; paternal asthma; gestational age at start of prenatal care; gestational fatty acid blood measurements; mode of delivery; season of birth; birth weight; duration of breastfeeding; child sex; offspring current wheezing, ever asthma; and eczema, offspring BMI; and age at BMI measurement. In the dataset of 20 imputations, we performed log linear regression or Poisson regression with robust variance to estimate RRs.

#### Mediation analysis

**Fig 1:**
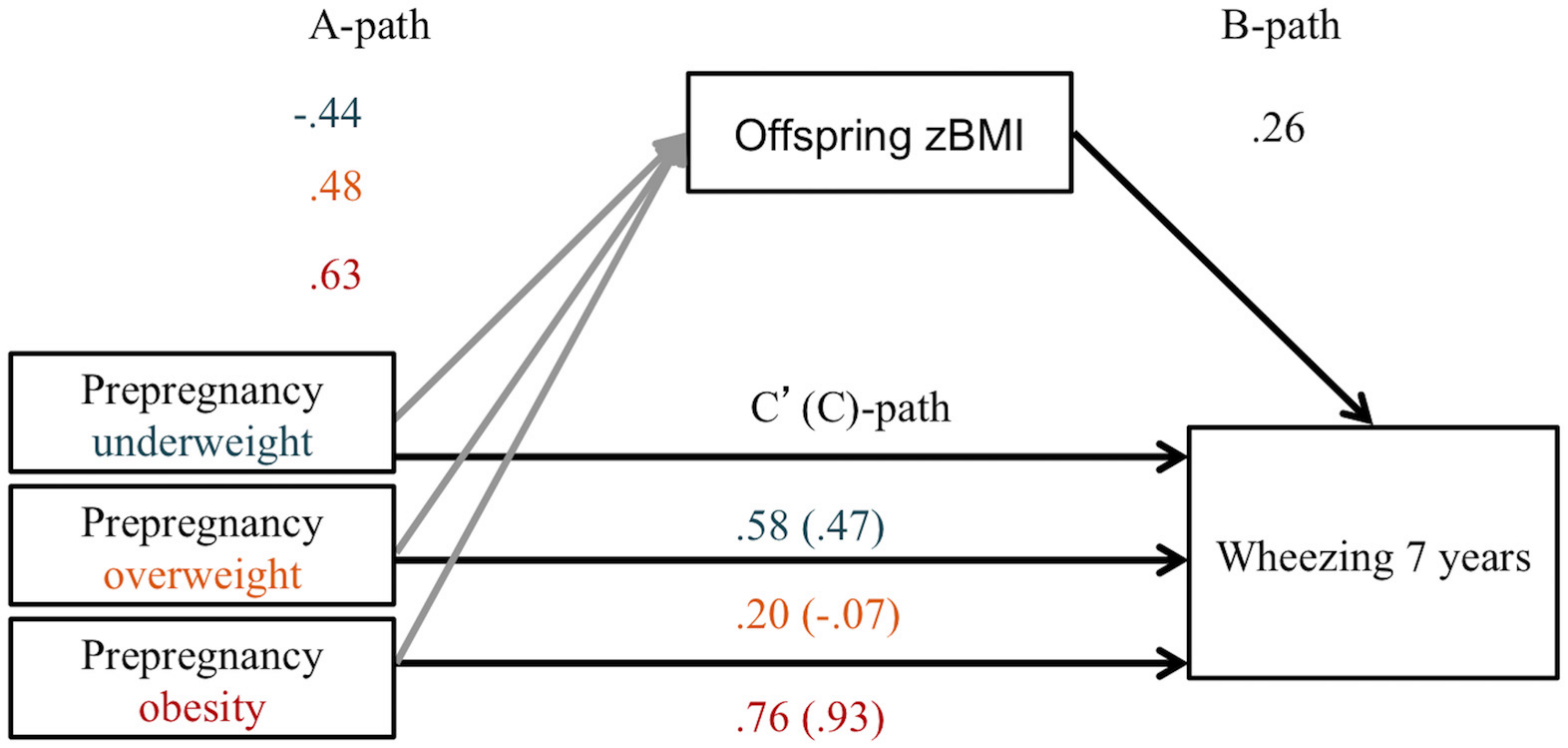
Indirect effect of pre-pregnancy BMI on current wheezing at age 7–8 years through offspring BMI z-score at age 5 years (n = 1781). Adjusted for: maternal age, parental asthma, ethnicity, maternal education. A-path: a-coefficient describing the change in offspring’s zBMI per BMI category in comparison to normal maternal BMI. B-path: b-coefficient describing change in offspring current wheezing risk per 1 point change in offspring zBMI when maternal BMI is constant. C’(C)-path: direct (total) effect.

We performed a simple mediator model [26, 35] with adjustment for confounders to test mediation by offspring BMI. This model uses linear regression to calculate the total, direct and indirect effect of maternal BMI categories on binary outcomes. We performed models for both outcomes. The mediator was offspring BMI z-score (zBMI) at age 5 years [31]. The indirect effect quantifies how much the risk in offspring of women that are underweight, overweight or obese, are estimated to differ compared from the control group (normal maternal BMI) as a result of the influence of maternal BMI on offspring BMI, which in turn influences the risk of current wheezing and ever asthma [26, 35]. The indirect effect is a quantitative measure and is a product of an a- and b-coefficient. The a-coefficient describes the change in offspring’s zBMI per maternal BMI category in comparison to normal maternal BMI. The b-coefficient describes the change in offspring asthma risk per 1 unit change in offspring’s zBMI when maternal BMI is constant (Fig 1). We calculated percentile bootstrap CIs for each indirect effect. Percentile bootstrap CIs are calculated to test statistical inference, while respecting non-normal sampling distribution. The indirect effects of k random samples of the original cases with replacement are calculated. From these k indirect effects, a lower and upper bound of a 95% are taken. If this confidence interval does not contain 0, the indirect effect is statistically significant. For a binary outcome, like asthma, this method quantifies the effect size of the indirect effect and direct effect on different scales, and so the total effect will not be equal to the sum of the direct and indirect effects. For this reason we do not report measures of effect size, like portions of mediation [26].

To test the interacting/moderating effect by parental asthma, we used a single moderator model. We calculated an interaction product term and tested statistical significance, controlling for potential confounding by western ethnicity; maternal education; and maternal age, in a linear regression model [26].

We used STATA version 12.0 for the analysis of demographics, multiple imputation and log linear regression models. We used SPSS version 20 with the PROCESS for SPSS v2.10 macro for the mediation and interaction analysis [26]. We considered a confidence interval that did not include 0 or a p < 0.05 (two sided) as statistically significantly.

## Results

### Study population

**Fig 2:**
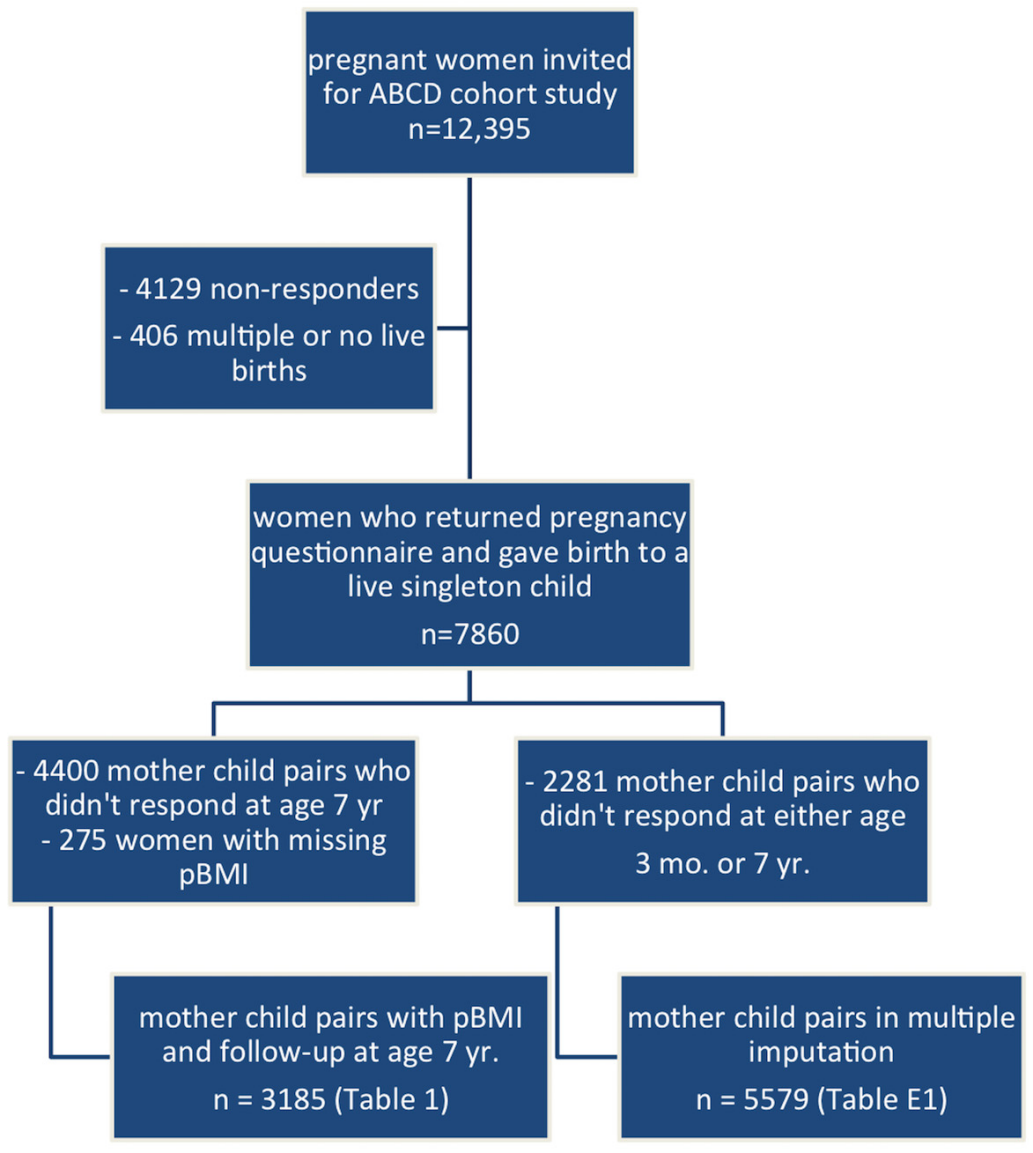
study outline.

Of all participating mothers in the ABCD study, 3185 women gave birth to a live singleton and responded to both the pregnancy questionnaire (reporting weight and height) and to the questionnaire at age 7–8 years. These women are included in the main analysis. In the multiple imputation analysis, we included all the 5579 mother-child pairs that responded to both the pregnancy questionnaire and the respiratory symptom questionnaires at either 3 months or 7–8 years (Fig 2).

Of the 3185 women, 4% were underweight, 15% were overweight and 4% were obese. Overweight and obese women were more often multiparous, of non-Western ethnicity, and had lower education. Their children were more often exposed to domestic smoking, more often born by caesarean-section, had a higher birth weight, and a higher BMI at age 5 years. In the groups of women with overweight and obesity were also higher rates of parental asthma. Gestational age at birth was lower in the underweight and obese groups (resp. 39.0 and 39.2 in comparison to 39.5 in mothers with a BMI between 18.5 and 25 kg/m^2^) (Table 1).

**Table 1:** Differences in demographics (mean or prevalence), as a function of prepregnancy BMI in mothers and their offspring (n = 3185) ^^^.

Maternal BMI (n (% of cohort))		BMI <25	BMI ≥ 25	All	n	p-value*
		n = 2580 (81%)	n = 605 (19%)	n = 3185 (100%)		
**Maternal age at pregnancy (years, mean (SD))**		32.2 (4)	31.9 (5)	32.1 (4)	3185	0.1
**Maternal prepregnancy BMI** (kg/m^2^, **mean (SD)**)		21.4 (2)	28.6 (4)	22.7 (4)	3185	<0.001
**Primiparous (n (%))**		1188 (46)	217 (36)	1405 (44)	3185	<0.001
**Ethnicity (n (%))**	Non-western	272 (11)	143 (24)	415 (13)	3185	<0.001
**Maternal education (years after primary school)**	0–5	221 (9)	130 (22)	351 (11)	3175	<0.001
	6–11	1172 (46)	289 (48)	1461 (46)		
	≥ 12	1182 (46)	181 (30)	1363 (43)		
**Tobacco exposure (n (%))**	Maternal smoking during pregnancy	193 (7)	48 (8)	241 (8)	3185	0.1
	Maternal smoking at either age 3mo/5y	413 (16)	102 (17)	515 (17)	3113	0.6
	Smoking in the house at either age 3mo/5y	227 (9)	84 (15)	311 (10)	3070	<0.001
**Parental asthma (n (%))**		169 (7)	60 (12)	229 (8)	2777	<0.01
**Caesarean section (n (%))**		300 (12)	100 (17)	400 (13)	3185	<0.01
**Birth weight (grams, mean (SD))**		3471 (523)	3559 (581)	3488 (535)	3182	<0.0001
**Gestational age at birth (weeks, mean (SD))**		39.5 (2)	39.5 (2)	39.5 (2)	2799	0.6
**Duration of breastfeeding (weeks, mean (SD))**		20.9 (15)	20.5 (18)	20.8 (16)	3130	0.7
**Offspring BMI at age 5 years (mean (SD))**		15.2 (1)	16.1 (2)	15.4 (1)	2259	<0.0001
**Offspring BMI z-score at age 5 years (mean (SD))**		-0.3 (1)	0.3 (1)	-0.18 (1)	2023	<0.0001

^^^ Mother-child pairs of life-singleton births with returned pregnancy-questionnaires, including maternal weight and height, and a response at 7 years. BMI: body mass index.

* p-value calculated with chi-square test, ttest or Mann-Whitney U test.

Comparing demographic information between mother-child pairs with follow up at 7 years to mother-child pairs with follow up at either 3 months or 7 years (n = 3460 versus n = 5579), we found that women with loss to follow-up at 7 years were more often of non-western ethnicity (20 versus 14.8%) and their children had a higher BMI z-score at age 5 years (mean score -0.07 (SD 0.02) versus -0.16 (SD 0.02)) (S1 Table).

### BMI and asthma

At the age of 7–8 years, 8% of the offspring ever had asthma and 7% had current wheezing. Maternal obesity was strongly associated with current wheezing (adjusted RR model 2: 2.15 (1.27, 3.64)) and ever asthma (RR model 2: 2.24 (1.45, 3.46)). Maternal overweight was not significantly associated with offspring current wheezing and ever asthma (Table 2). The association between maternal obesity and offspring current wheezing and ever asthma was in the same direction in the imputed dataset but attenuated (RR model 2 in obese group for current wheezing 1.58 (95% CI 1.01, 2.49) and for ever asthma 1.70 (1.17, 2.48)) (Table 2, S2 Table). There was no significant interaction by parental asthma in the association between maternal BMI and current wheezing (p = 0.34) or ever asthma (p = 0.41).

**Table 2:** RRs of respiratory outcomes at offspring age 7-8years in relation to maternal BMI (n = 3185).

		Prevalance (n(%))	Crude RR (95% CI)	Adjusted RR Model 1 (95% CI) ‡	Adjusted RR Model 2 (95% CI)^	Adjusted RR Model 2 imputed data (95% CI)^ *
**Current wheezing in past 12 months**	Underweight	n = 10 (7%)	1.15 (0.62, 2.12)	1.34 (0.70, 2.56)	1.32 (0.69, 2.53)	0.99 (0.58, 1.68
	Normal weight	n = 151 (6%)	Reference	Reference	Reference	Reference
	Overweight	n = 27 (6%)	0.93 (0.62, 1.38)	0.91 (0.58, 1.43)	0.90 (0.57, 1.42)	1.11 (0.82, 1.51)
	Obese	n = 17 (13%)	2.09 (1.30, 3.33)	2.16 (1.28, 3.64)	2.15 (1.27, 3.64)	1.58 (1.01, 2.49)
**Ever asthma diagnosed by medical doctor**	Underweight	n = 15 (11%)	1.64 (0.99, 2.71)	1.47 (0.84, 2.59)	1.42 (0.81, 2.50)	1.14 (0.72, 1.81)
	Normal weight	n = 157 (7%)	Reference	Reference	Reference	Reference
	Overweight	n = 40 (9%)	1.33 (0.96, 1.86)	1.42 (0.99, 2.02)	1.38 (0.97, 1.96)	1.25 (0.94, 1.65)
	Obese	n = 24 (18%)	2.72 (1.84, 4.03)	2.32 (1.49, 3.61)	2.24 (1.45, 3.46)	1.70 (1.17, 2.48)

^‡^ adjusted for: length of education (categorical); maternal age; western ethnicity; and parental asthma.

^^^ adjusted for: duration of breastfeeding; length of education (categorical); maternal age; western ethnicity: parental asthma; parity; mode of delivery; smoking during pregnancy; and domestic smoking

* also adjusted for offspring zBMI

RR: risk ratio; CI: confidence interval

### Mediation by offspring BMI

**Fig 3:**
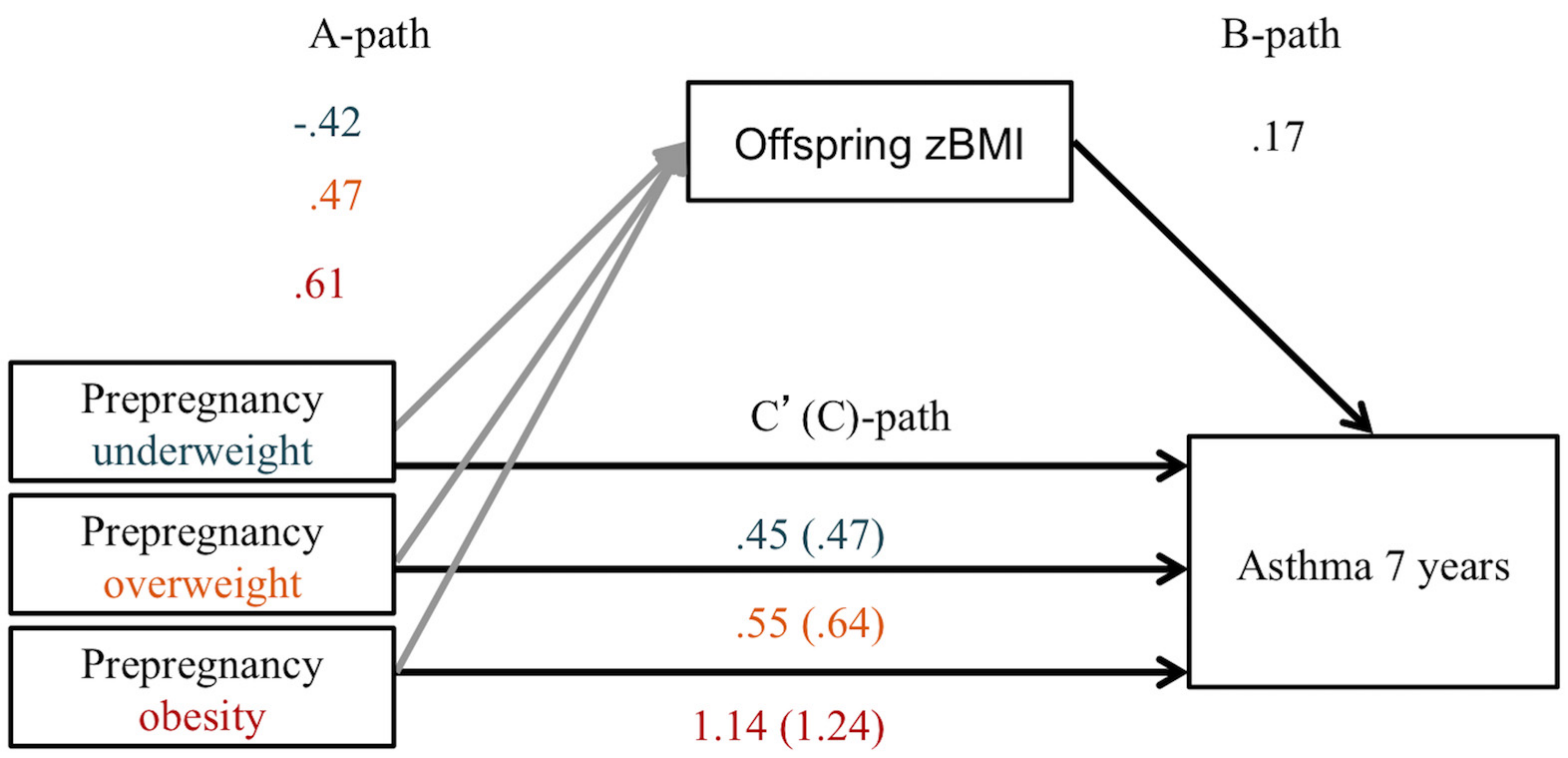
Indirect effect of pre-pregnancy BMI on ever asthma at age 7–8 years through offspring BMI z-score at age 5 years (n = 1787). Adjusted for: maternal age, parental asthma, ethnicity, maternal education, A-path: a-coefficient describing the change in offspring’s zBMI per BMI category in comparison to normal maternal BMI. B-path: b-coefficient describing change in offspring asthma risk per 1 point change in offspring zBMI when maternal BMI is constant. C’(C)-path: direct (total) effect.

In a mediator model, maternal BMI appeared to indirectly influence risk of current wheezing at the age of 7–8 years through its effect on offspring BMI. As can be seen in Fig 1, children of overweight and obese mothers had a higher BMI z-score (a-coefficient = 0.48 and 0.63) and children with a higher BMI z-score had a higher risk of current wheezing (b-coefficient = 0.26) (Fig 1). A percentile bootstrap CI for the indirect effect of overweight or obesity was entirely above zero (overweight: indirect effect (ab) = 0.124 (CI = 0.020, 0.243); obesity: indirect effect (ab) = 0.164 (0.024, 0.348) (Table 3). Obesity also influenced the risk of current wheezing directly in our model (direct effect (c’) = 1.16 (0.53, 1.80), suggesting that other factors are also playing a role in the causal relationship between maternal obesity and offspring current wheezing (Fig 1). In a separate model with the outcome ever asthma at age 7–8 years, offspring BMI was not a significant mediator (Fig 3 and Table 3).

**Table 3:** Indirect effect of z-score BMI offspring at age 5 years by maternal BMI category.

Outcome, by pBMI		Indirect effect (Boot CI) ^
Current wheezing at age 7–8 years	Underweight	-0.112 (-0.238, -0.017)
	Normal	Reference
	Overweight	0.124 (0.020, 0.243)
	Obese	0.163 (0.024, 0.348)
Ever asthma at age 7–8 years	Underweight	-0.074 (-0.172, 0.009)
	Normal	Reference
	Overweight	0.082 (-0.010, 0.179)
	Obese	0.103 (-0.013, 0.250)

BMI: body mass index; CI: confidence interval

^^^ adjusted for: length of education (categorical); maternal age; western ethnicity; and parental asthma.

## Discussion

In this large prospective birth cohort, maternal BMI had a direct and indirect (mediated) effect through offspring BMI on the risk of current wheezing at age 7–8 years, but only a direct effect on the risk of ever asthma. There was no interaction between parental asthma and maternal obesity in the association with current wheezing or ever asthma.

In contribution to the existing literature of an association between maternal pre-pregnancy BMI and respiratory outcomes in the offspring, we tested the mediating effect of the child’s own BMI by testing the mediated effect computed as the product of regression coefficient estimates. We identified offspring BMI as a mediating factor for current wheezing, meaning that maternal overweight and obesity are associated with a higher offspring BMI, which in turn increases the risk for current wheezing. Offspring BMI might be associated with maternal BMI due to shared environmental factors or fetal programming [22, 23]. Increased offspring BMI is epidemiologically being linked to offspring ever asthma [21, 36–38]. A significant remaining direct effect of maternal BMI on offspring current wheezing implies that there are other causal pathways that play a role as well. Perhaps these pathways are even more important in the association with offspring ever asthma, for which offspring BMI was not a significant mediator in our analysis. Possible factors that can also be investigated are dietary habits of mother and child; markers of atopy, fitness and autonomic functions and environmental factors [12, 19, 22–25].

Our study confirms the positive relationship between maternal pre-pregnancy BMI and current wheezing and ever asthma at age 7–8 years that has been found by a recent meta-analysis [14]. This meta-analysis also concluded that maternal asthma causes a borderline significant effect modification/interaction, based a more pronounced increase in the risk of childhood asthma when the prevalence of maternal asthma was lower. We did not find effect modification by parental asthma by calculating and testing an interaction product term with a single moderator model.

The current study has important strengths. The ABCD study provides a unique opportunity to investigate the mechanism by which maternal obesity affects offspring respiratory outcomes in a large urban multiethnic cohort. We have detailed socio demographic, lifestyle and cardio metabolic information on mothers and their offspring. Outcome measurements are obtained with common validated questionnaires, which enable comparison with other studies. In this analysis we tested mediation by offspring BMI and effect modification/interaction by parental asthma. By using this method we can estimate and test hypotheses about the two pathways of influence through which maternal BMI carries its effect on offspring outcomes: one direct pathway and one indirect through childhood BMI. We quantify and test the indirect effect, not depending on a statistically significant total effect. For all these reasons, this new mediation analysis is favorable over the older causal steps approach, also known as the Baron and Kenny method, and by our knowledge, we are the first to perform it in birth-cohort study [39].

The current study also has limitations that need to be addressed. Outcome measurements are all from self-reported questionnaires in our study, which may lead to misclassification or underreporting. We have reduced misclassification by using asthma diagnosed by a physician at age 7–8 years, reported by parental questionnaire answers, which is frequently used by others [7, 10, 11], The exposure measurement, maternal pre-pregnancy BMI, is self-reported during early pregnancy and is likely underreported in overweight and obese women, which might have attenuated our estimates toward the null. The short time-lapse (12 weeks) between self-weighing before pregnancy and the time of recall during the questionnaire might further reduce recall bias, compared to studies that have a one-year time lapse between self-weighing and recall by the mother. We did not collect data on gestational weight gain, a potential confounder in the association between maternal BMI and respiratory outcomes. Our cohort represented all different socio-economic and ethnic groups at baseline and 3 month follow-up, with the help of multi-lingual questionnaires and phone call reminders. As these extra services were not available for the questionnaire at age 7 years, relatively more Western mothers responded at this time. Differential loss to follow up was addressed by multiple imputation analysis. With multiple imputations we were able to impute missing variables for all mother child pairs that returned the pregnancy questionnaire and one outcome questionnaire, a cohort with 20% non-Western mothers. Selective non-response and selection bias in the ABCD study at the time of study enrollment have been investigated and reported by Tromp et al. Their results indicated that selective non-response was present, but selection bias was acceptably low for adverse pregnancy outcomes [40].

Our findings have clinical implications for prenatal and child health care. Pre-pregnancy obesity can be prevented by parental counseling before conception and by raising public awareness. Offspring obesity can be a delicate subject during wellbeing child visits, but research data on its short- and long-term effects could be useful in motivational counseling. Reduction of offspring BMI in children of obese or overweight mothers might reduce the risk of offspring current wheezing, according to our mediation analysis results. Our results support the theory that interventions should be offered to all overweight or obese children, especially to high-risk children that were born from mothers with pre-pregnancy obesity or overweight.

### Conclusions

We found associations between pre-pregnancy obesity and higher risks of ever asthma and current wheezing at age 7–8 years. Offspring BMI is a mediator in the effect of overweight and obesity on current wheezing. Future research needs to be done to determine how interventions before conception and during early childhood can increase respiratory health effectively.

## Supporting Information

S1 Fig DAG for prepregnancy BMI. Red arrows: open biasing paths; green arrows: open causal paths; pink oval: ancestor of exposure; blue ovals: ancestor of outcome.
(TIF)

S1 Table Demographic differences (mean or prevalence), between mother-child pairs with follow up at 7 years or multiple imputed data for mother-child pairs with follow up at either 3 months or 7 years. (DOC)

S2 Table Risk Ratio’s of respiratory outcomes in offspring in relation to prepregnancy BMI from imputed dataset (n = 5579). † Risk ratios estimated by logistic regression; ¥ Risk ratios estimated by Poisson Regression; ‡ adjusted for length of education (categorical), maternal age, western ethnicity, and parental asthma; ^ adjusted for: duration of breastfeeding; length of education (categorical); maternal age; western ethnicity; parental asthma; parity; mode of delivery; smoking during pregnancy; and domestic smoking.
(DOC)
