# Supplementary figures and images for: A Study on Mediation by Offspring BMI in the Association between Maternal Obesity and Child Respiratory Outcomes in the Amsterdam Born and Their Development Study Cohort
Source: PLoS One. 2015 Oct 20;10(10):e0140641. doi: 10.1371/journal.pone.0140641 (PMC4618476; doi:10.1371/journal.pone.0140641)

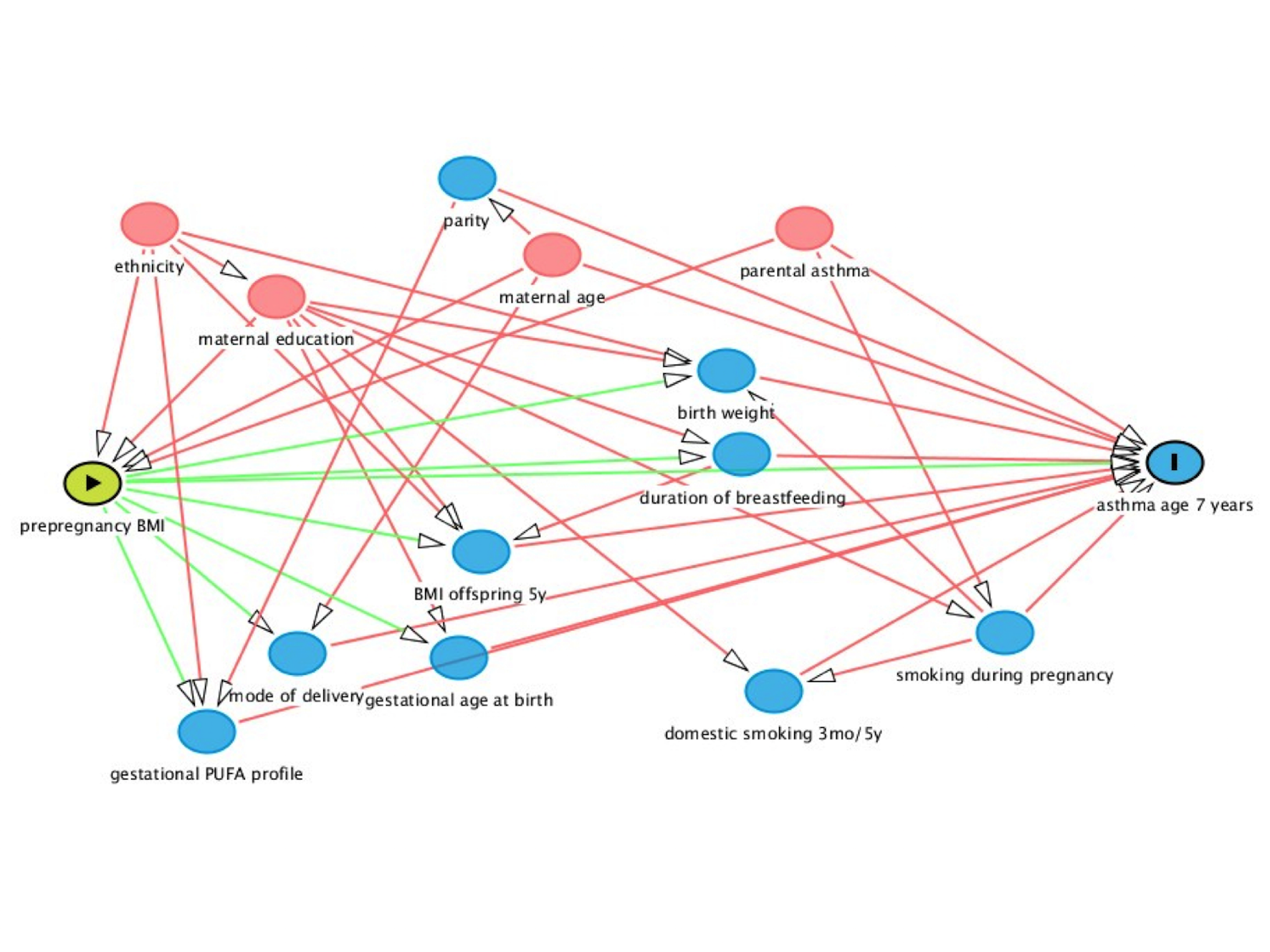

Supplement: S1 Fig — Red arrows: open biasing paths; green arrows: open causal paths; pink oval: ancestor of exposure; blue ovals: ancestor of outcome. (TIF) [file pone.0140641.s001.tif]
