# Supplementary material for: A Study on Mediation by Offspring BMI in the Association between Maternal Obesity and Child Respiratory Outcomes in the Amsterdam Born and Their Development Study Cohort
Source: PLoS One. 2015 Oct 20;10(10):e0140641. doi: 10.1371/journal.pone.0140641 (PMC4618476; doi:10.1371/journal.pone.0140641)
Supplement: S1 Table — (DOC) [file pone.0140641.s002.doc]

**S1 table: Demographic differences (mean or prevalence), between mother-child pairs with follow up at 7 years or multiple imputed data for mother-child pairs with follow up at either 3 months or 7** years.

|  | Mothers who returned questionnaire at age 7 years.  n= 3185 | Imputed dataset with mothers who returned questionnaire at age 3 months or 7 years; n= 5579. |
| --- | --- | --- |
| **Maternal age at pregnancy (years, mean (SE))** | 32.1 (0.08) | 31.4 (0.06) |
| **Primiparous (%)** | 44 | 42 |
| **Prepregnancy BMI (kg/m^2^, mean (SE))** | 22.7 (0.07) | 22.9 (0.05) |
| **Ethnicity (%)** Non-Western | 13 | 20 |
| **Maternal education (years after primary school) (mean (SE))** | 10 (0.06) | 9.5 (0.05) |
| **Tobacco exposure (%)**  Maternal smoking during pregnancy  Maternal smoking at either age 3mo/5y  Smoking in the house at age 3mo/5y | 8  17  10 | 9  20  14 |
| **Parental asthma (%)** | 8 | 9 |
| **C-section (%)** | 13 | 13 |
| **Birth weight (grams, mean (SE))** | 3488 (9) | 3466 (7) |
| **Gestational age at birth  (weeks, mean (SE))** | 39.5 (0.03) | 39.5 (0.02) |
| **Duration of breastfeeding  (weeks, mean (SE))** | 20.8 (0.3) | 19.3 (0.2) |
| **BMI at age 5 years (mean (SE))** | 15.4 (0.03) | 15.5 (0.03) |
| **BMI z-score at age 5 years (mean (SE))** | -0.18 (0.02) | -0.07 (0.02) |
