# Supplementary material for: A Study on Mediation by Offspring BMI in the Association between Maternal Obesity and Child Respiratory Outcomes in the Amsterdam Born and Their Development Study Cohort
Source: PLoS One. 2015 Oct 20;10(10):e0140641. doi: 10.1371/journal.pone.0140641 (PMC4618476; doi:10.1371/journal.pone.0140641)
Supplement: S2 Table — † Risk ratios estimated by logistic regression; ¥ Risk ratios estimated by Poisson Regression; ‡ adjusted for length of education (categorical), maternal age, western ethnicity, and parental asthma; ^ adjusted for: duration of breastfeeding; length of education (categorical); maternal age; western ethnicity; parental asthma; parity; mode of delivery; smoking during pregnancy; and domestic smoking. (DOC) [file pone.0140641.s003.doc]

**S2 t**able: Risk Ratio’s of respiratory outcomes in offspring in relation to prepregnancy BMI from imputed dataset (n=5579)

|  | Crude RR (95% CI) | RR Model 1 (95% CI) ‡ | RR Model 2 (95% CI)^ |
| --- | --- | --- | --- |
|  |  |  |  |
| **Wheezing in past 12 months at age 7 years †**  Underweight  Normal weight  Overweight  Obese | 1.03 (0.61, 1.75)  Ref  1.23 (0.90, 1.68)  1.91 (1.24, 2.95) | 0.99 (0.58, 1.70)  Ref  1.13 (0.83, 1.53)  1.63 (1.04, 2.54) | 0.99 (0.58, 1.68  Ref  1.11 (0.82, 1.51)  1.58 (1.01, 2.49) |
| **Ever asthma diagnosed by medical doctor ¥**  Normal weight  Underweight  Overweight  Obese | 1.22 (0.77, 1.93)  Ref  1.45 (1.10, 1.92)  2.25 (1.58, 3.21) | 1.14 (0.72, 1.81)  Ref  1.29 (0.97, 1.71)  1.83 (1.25, 2.66) | 1.14 (0.72, 1.81)  Ref  1.25 (0.94, 1.65)  1.70 (1.17, 2.48) |

† Risk ratios estimated by logistic regression; ¥ Risk ratios estimated by Poisson Regression;

‡ adjusted for length of education (categorical), maternal age, western ethnicity, and parental asthma;

^ adjusted for: duration of breastfeeding; length of education (categorical); maternal age; western ethnicity;

parental asthma; parity; mode of delivery; smoking during pregnancy; and domestic smoking.
